# Empowering change: A cross-sectional study on quality of life in deinfibulated women at Saudi Teaching Hospital, Kassala-Sudan 2023–2024

**DOI:** 10.1371/journal.pgph.0004096

**Published:** 2025-02-14

**Authors:** Wafa Y. Ahmed, Lina S. Altayib, Alaa T. Omer

**Affiliations:** 1 Faculty of Medicine, University of Khartoum, Khartoum, Sudan; 2 Education and Development Center EDC - University of Khartoum, Khartoum, Sudan; Centre of Biomedical Ethics and Culture, PAKISTAN

## Abstract

Deinfibulation [opening the scar of infibulation] is a minor operation involving a vertical incision to the scar to expose the urethra and vagina. Deinfibulation may improve the quality of life for infibulated women. The quality of life after deinfibulation has been sparsely investigated. This study aims to fill this gap by evaluating the quality of life in deinfibulated women, examining the effects of deinfibulation on the urogenital system, and identifying the reasons for not performing reinfibulation. The study aimed to evaluate the quality of life in women who have undergone deinfibulation and to assess the impact of deinfibulation on the urogenital system. A descriptive cross-sectional study was conducted at Saudi Teaching Hospital in Kassala, involving 73 women who previously underwent infibulation and subsequently underwent deinfibulation. Data collection included sociodemographic information, gynecological and obstetric history, reasons for deinfibulation, reasons behind not performing reinfibulation, and assessments of complications such as urinary tract infections and sexual symptoms. Among the participants, 54.8% had female genital mutilation type III FGM, while 45.2% had female genital mutilation type IV. The percentage of women with regular menstrual cycles increased to 100% after deinfibulation. Dysmenorrhea was reported by only 9.6% of participants after deinfibulation. The prevalence of obstructed labor decreased to 1.4%. Episiotomy was performed on all women before deinfibulation, with 78.1% reporting it after deinfibulation. None of the participants had blood loss after deinfibulation. Before deinfibulation, 24.7% of women reported various sexual symptoms, while urinary tract infections were more prevalent at 87.8%. The pain was the primary reason for not undergoing reinfibulation 50.7%, and vaginal delivery was the most common reason for deinfibulation 97.3%. Deinfibulation has the potential to enhance the quality of life for women who have undergone infibulation and demonstrates positive effects on the urogenital system.

## Introduction

Female genital mutilation [FGM] refers to a range of procedures that involve the partial or complete removal of the external female genitalia or other forms of injury to the female genital organs for non-medical reasons [1]. As of 2024, more than 230 million girls and women worldwide have undergone FGM, with Africa being the region with the highest prevalence. Over 144 million cases of FGM have been reported in Africa alone. Sudan, in particular, has an estimated prevalence rate of 87% among girls and women, according to data from the UNICEF global databases [2]. Female Genital Mutilation (FGM), is practiced in Sudan primarily due to deeply rooted beliefs about purity, modesty, and marriageability. Historically, it has been tied to societal expectations of controlling female sexuality and ensuring marital prospects. Efforts to abolish female genital mutilation (FGM), in Sudan have involved a combination of legal, educational, and grassroots initiatives. Despite these efforts, challenges remain, including deep-rooted cultural beliefs, social pressure, resistance from some communities, and limited resources for enforcement of the new laws [3].

The World Health Organization [WHO] has classified FGM into four main types [Table 1] [1].

**Table 1:** WHO Classification of Female Genital Mutilation (FGM) Types, Definitions and Categories.

Type	Details
**Type I**	Excision of prepuce and part or all of clitoris.
**Type II**	Partial or total removal of the clitoral glans and the labia minora with or without removal of the labia majora.
**Type III**	Infibulation, cutting and repositioning the labia minora, or labia majora, sometimes through stitching, with or without removal of the clitoral prepuce/clitoral hood and glans.
**Type IV**	Pricking, piercing, incision, stretching, scraping, or other harming procedures on clitoris and or labia.

While another local classification by a different author [4] groups the main types into two broader categories. The first category includes clitoridectomies (removal of the clitoris), which encompass Types I and II. The second category consists of infibulations, which include Types III and IV. Type I involves the removal of the clitoris, while Type II involves clitoridectomy along with partial or total excision of the labia minora. Type III, also known as modified infibulation or intermediate circumcision, is a milder form of infibulation where the incision of the labia majora is limited to the upper two-thirds, leaving a larger posterior opening. Total infibulation, also known as pharaonic circumcision, is Type IV and involves the removal of the clitoris and labia minora, as well as the incision of the labia majora. The raw surfaces are then stitched together to cover the urethra and vaginal entrance, leaving a small opening for urine and menstrual blood to pass through.

FGM can lead to immediate complications such as severe pain, bleeding, infections, urinary problems, wound healing issues, damage to surrounding genital tissue, shock, and even death. In the long term, it can result in urinary, vaginal, menstrual, and sexual problems, as well as an increased risk of complications during childbirth [1].

Deinfibulation, a minor surgical procedure, involves making a vertical incision in the scar tissue to expose the urethra and vagina. This procedure is recommended for infibulated women who are pregnant or experiencing long-term complications such as dysmenorrhea (Painful menstruation), dyspareunia (Painful coitus), apareunia (inability to perform sexual intercourse), or chronic vaginal and urinary infections to alleviate or resolve these issues [5–8]. It is important to note that tight infibulation can pose significant risks to both the mother and the fetus. In cases where infibulation is present, deinfibulation is often necessary to allow for the safe passage of the fetal head during childbirth and may also be required for the first sexual intercourse. While a physician or midwife can perform the procedure, in situations where trained practitioners are not available, the infibulation may be opened by the husband or a female relative using any sharp object at hand [9].

Failure to perform deinfibulation can put women with infibulation [type III and IV FGM] and their unborn babies at serious risk [4]. The green-top guidelines of the Royal College recommend deinfibulation if the vaginal opening is not sufficiently wide to allow for normal urinary and menstrual flow, comfortable sexual intercourse, safe vaginal delivery, cervical smears, sexual health screens, and gynecological surgery [10]. In practice, deinfibulation is necessary for women with infibulation as it narrows the vaginal introitus. It is advisable to recommend deinfibulation during the antenatal period, although some women may prefer to undergo the procedure during labor, as is the customary practice in certain countries where FGM is prevalent [10].

It should be emphasized that deinfibulation does not provide a complete solution for FGM, as it is incapable of reversing the harm caused by the initial mutilation. Instead, it is typically carried out to alleviate certain health issues that may result from FGM, including difficulties with urination, menstruation, sexual intercourse, and childbirth.

The World Health Organization [WHO] defines the quality of life [QOL] as “an individual’s perception of their position in life in the context of the culture and value systems in which they live and about their goals, expectations, standards, and concerns” [11].

In Sudan, there have been no studies conducted on the impact of deinfibulation on overall quality of life. Therefore, this research aims to fill this gap by evaluating the quality of life in deinfibulated women, examining the effects of deinfibulation on the urogenital system [including urinary tract infections UTI, menstrual flow, sexual life, and delivery], and identifying the reasons for not performing reinfibulation.

## Methods

### Study design, setting, and period

From November 5th, 2023 to July 3rd, 2024, a hospital-based descriptive cross-sectional study was conducted at Saudi Teaching Hospital in Kassala town. Kassala, located in Sudan, is one of the 18 states in the country. With an area spanning 36,710 km², it is home to an estimated population of around 2,519,071 as of 2018. Serving as the state capital [12]. Kassala is supported by the Saudi Teaching Hospital, the sole facility specializing in obstetric and gynecological care in the region. This hospital plays a crucial role in providing antenatal and delivery services to the state’s population and is a generous contribution from the Kingdom of Saudi Arabia [13].

### Study population

Multiparous (a female who has given birth two, three, or four times) women who had undergone deinfibulation, presenting with FGM type III or IV, and seeking care at Saudi Teaching Hospital during the study period.

Primiparous (a female who has given birth once) and those undergoing reinfibulation were excluded from the analysis to facilitate a comparison between childbirth outcomes pre- and post-deinfibulation.

### Sample size

The study sample [non-probability simple random sampling] was calculated using the Cochran formula:


$$\text{n}=\text{Z}²\text{P}\left(1-\text{P}\right)÷\text{d}²$$

with assumptions of: 8% level of precision [d]=0.08

95% confidence level [Z]=1.96

Population proportion [P]=87%=0.87


q=1-p=0.13

Sample size [n]=67

Calculated Sample size was 67, We took a sample of 73.

### Sampling technique

A non-probability convenience sampling of 73 women was conducted.

### Data collection method

#### Instruments.

An author-designed questionnaire contained 32 closed and open-ended questions in five domains. This included sociodemographic data, gynecological, obstetric history, deinfibulation purpose/reason behind not performing re-infibulation, and also assessing complications such as UTI and sexual symptoms. The questionnaire was reviewed by expert researcher, obstetrician, and statistician to check for reliability.

The data collection tool was administered in Arabic to ensure that participants could understand the context.

#### Technique.

The data was collected during the period from November 5th to November 26th through face-to-face interviews by the authors themselves.

### Data analysis

Data were initially entered and cleaned using Microsoft Excel, then analyzed using Statistical Package for Social Sciences [SPSS] version 20. Descriptive statistics were used, and categorical data was presented in frequencies [n] and percentages [%].

### Ethical consideration

Ethical approval was obtained at 25th Sep 2023, from the community department at the University of Khartoum under ID number COMMED 2023-95-1, and permission was obtained from the Saudi Teaching Hospital administration. Informed consent was obtained from each participant. Participants under the age of 18 are considered emancipated minors because they are married. While this might not be the norm in other places, Sudan’s social norms and attitudes frame married girls as adults. This classification extends to healthcare settings, where this girls receive adult-level care and are often managed outside of the pediatric department. Each respondent was informed about the purpose of the study, and all data obtained from them was kept confidential.

## Results

### Sociodemographic characteristics of study participants

A total of 73 women participated in this study; the most common age group was 18–25 years 39.7%, followed by 26–32 years 27.4%.

More than half of the respondents were residents of urban areas 56.2%, while 43.8% were rural residents. The majority (76.7%) had a moderate social class (middle-income group that typically has access to essential services but may not have significant disposable income). Regarding occupation, almost all women who participated were housewives 97.3% [Table 2].

**Table 2:** Baseline characteristics of study participants, (N = 73).

Characteristics	Frequency	Percent
**Age group:**		
<18 years	6	8.2
18–25 years	29	39.7
26–32 years	20	27.4
>32 years	18	24.7
**Residence:**		
Rural	32	43.8
Urban	41	56.2
**Social class:**		
Low	14	19.2
Moderate	56	76.7
High	3	4.1
**Occupation:**		
Housewife	71	97.3
Employee	1	1.4
Doctor	1	1.4

### FGM-related information

**Fig 1:**
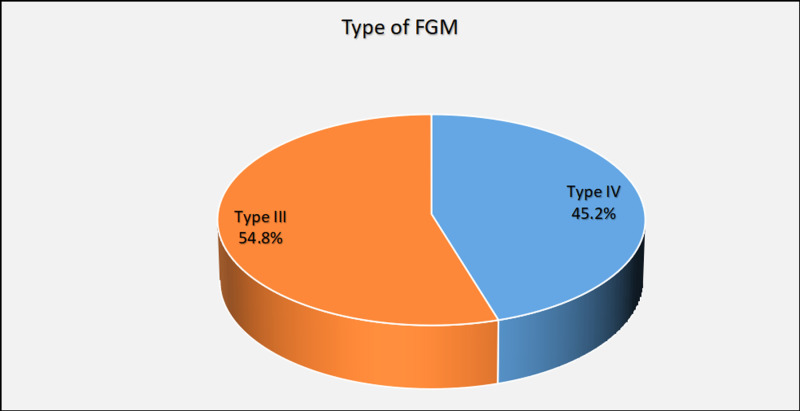
Shows type of FGM which patient underwent, (N = 73). This pie chart showed type of FGM patient underwent, FGM type III was more common among participants than type IV.

**Fig 2:**
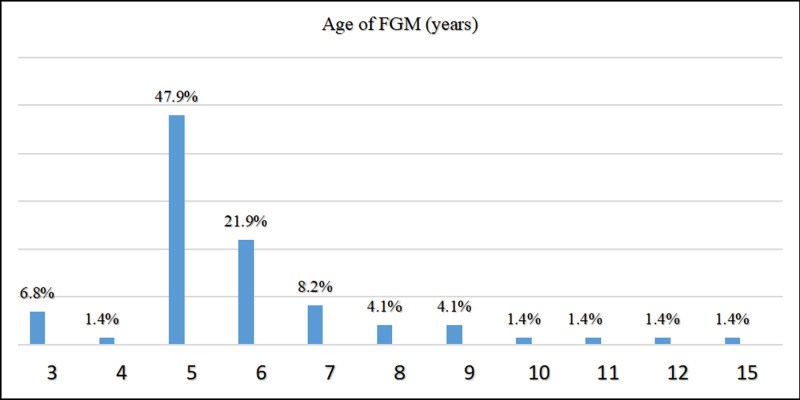
Age at which participants underwent FGM, (N = 73). This figure showed the common age at which participants underwent FGM was 5 years.

FGM type III was more common among participants than type IV 54.8% and 45.2%, respectively [Fig 1]. The age of 5 years was the most common age for FGM 47.9% [Fig 2].

### Gynecological and obstetric history and outcome before and after deinfibulation

Before deinfibulation, 83.6 percent of participants had regular menses; this percentage increased to 100% after deinfibulation. Before deinfibulation, 37% complained of dysmenorrhea, while only 9.6% experienced it after deinfibulation. Before deinfibulation 95.9%, delivered through spontaneous vaginal delivery, the percent decreased to 90.4% after deinfibulation. The prevalence of obstructed labor decreased from 65.8% before deinfibulation to 1.4% after it. All women underwent episiotomy before deinfibulation, while 78.1 percent reported it after deinfibulation. All of the women 100% had an intact perineum during delivery after deinfibulation, compared to 91.8 percent before it. None of the participants had blood loss during delivery after deinfibulation [Table 3].

**Table 3:** Gynecological and obstetric history and outcome before and after deinfibulation, (N = 73).

Gynaecological and obstetric history and outcome	Answer	Before deinfibulation		After deinfibulation	
		N	%	N	%
**Regularity of menses**	Regular	61	83.6	73	100.0
	Irregular	12	16.4	0	0
**Dysmenorrhea**	Yes	27	37.0	7	9.6
	No	46	63.0	66	90.4
**Mood of delivery**	SVD	70	95.9	66	90.4
	CS	3	4.1	7	9.6
**Obstructed labor**	Yes	48	65.8	1	1.4
	No	25	34.2	72	98.6
**Episiotomy**	Yes	73	100	57	78.1
	No	0	0	16	21.9
**Intact perineum**	Yes	67	91.8	73	100.0
	No	6	8.2	0	0
**Blood loss** **(During delivery)**	Yes	8	11.0	0	0
	No	65	89.0	73	100.0

### Sexual symptoms

Over two-thirds of participants, specifically 74%, reported no lack of interest in sexual activity. In contrast, 26% indicated they had experienced such a lack before deinfibulation. Additionally, 75.3% of participants reported not facing any issues. Conversely, 24.7% acknowledged having encountered these sexual symptoms before deinfibulation [Table 4].

**Table 4:** Sexual symptoms, (N = 73).

Statement	Answer	Frequency	Percent
**Lack of interest**	Never	54	74.0
	Before deinfibulation	19	26.0
**Lack of lubrication**	Never	55	75.3
	Before deinfibulation	18	24.7
**Difficulty becoming aroused**	Never	55	75.3
	Before deinfibulation	18	24.7
**Taking a long time to become aroused**	Never	55	75.3
	Before deinfibulation	18	24.7
**Pain or discomfort during sexual activity**	Never	55	75.3
	Before deinfibulation	18	24.7
**Difficulty achieving an orgasm (release of water)**	Never	55	75.3
	Before deinfibulation	18	24.7

### History of UTI

In this study, 71.2 percent of women had UTIs before, and all of them 100% were diagnosed by a doctor. The majority 90.4% had UTIs both before and after deinfibulation. Majority of the cases, 87.8% showed that before deinfibulation UTI was more frequent than after deinfibulation UTI [Table 5].

**Table 5:** History of UTI.

Statement	Answer	Frequency	Precent
**Have you ever experienced a UTI?**	Yes	52	71.2
	No	21	28.8
**Were you diagnosed by a doctor?**	Yes	52	100.0
	No	0	0
**When?**	After deinfibulation	2	3.8
	Before deinfibulation	3	5.8
	Both	47	90.4
**If both, which was the more frequent?**	After deinfibulation	6	12.2
	Before deinfibulation	43	87.8

### Reasons behind not performing reinfibulation and purpose of deinfibulation

**Fig 3:**
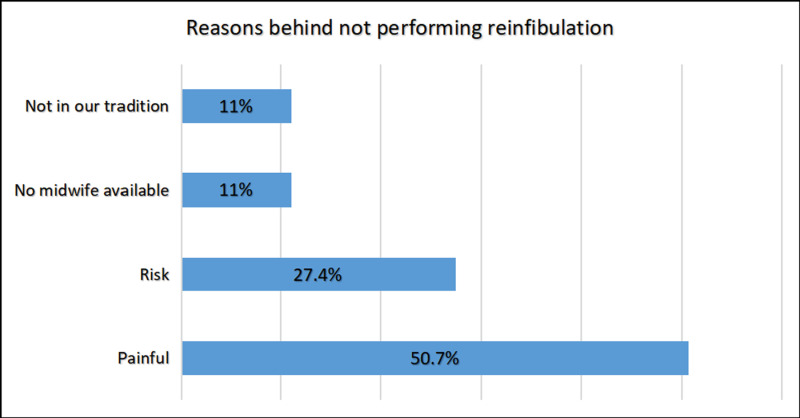
Reasons behind not performing reinfibulation, (N = 73). This figure showed Pain was the most popular reason behind not performing reinfibulation.

**Fig 4:**
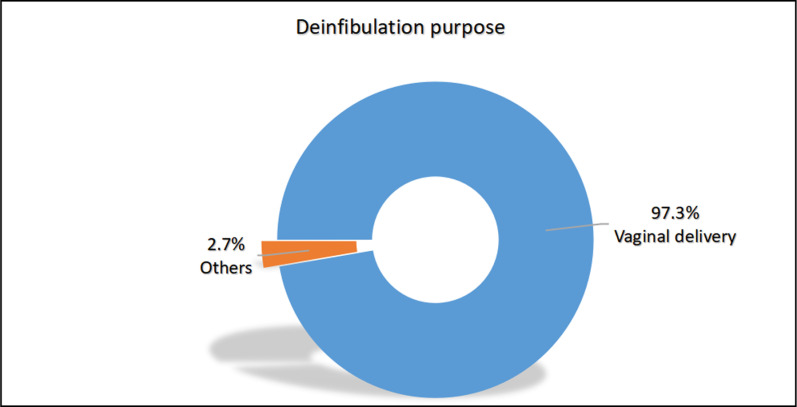
Purpose of deinfibulation, (N = 73). This figure showed vaginal delivery was the most prevalent purpose of deinfibulation among participants.

Pain was the most popular reason behind not performing reinfibulation 50.7%, while vaginal delivery was the most prevalent purpose of deinfibulation among participants 97.3% [Figs 3, 4].

### Discussion

The prevalent age group observed in the study falls within the range of 18–25 years. This is attributed to the fact that over sixty percent of Sudanese women marry at or before the age of eighteen, according to UNICEF [14]. More than half of the participants were from urban areas. Due to the fact that rural women opt to give birth at home instead of seeking medical assistance at hospitals, nearly half of rural community women chose to deliver at home with traditional birth assistants or no assistance [15]. FGM type III [intermediate] is more prevalent compared to type IV [Pharaonic], albeit with a minor variation. This can be attributed to the fact that the Pharaonic type of FGM has been abandoned and is now only found among older individuals or specific tribes like Beja, Beni Amir and Hadandawa [16]. Over the years, there has been a decline in Pharaonic circumcision, and a greater number of people have opted for the Sunnah, or intermediate type [17]. The age at which FGM is most often performed is 5 years, which is the median age for FGM in Sudan, according to Satti, A. et al. [18].

Additionally, we encountered a case where FGM was performed at the age of 15, which is considered relatively late. However, in this particular instance, the procedure was carried out at the insistence of the participant’s mother-in-law before the marriage was consummated. According to Toubia, FGM may be postponed until just before marriage or even after the birth of the first child [4].

The findings of the present study showed that the rate of menstrual regularity has increased significantly. We hypothesized that deinfibulation can help alleviate some of the complications associated with FGM, including menstrual irregularities. By removing any obstruction caused by scar tissue or closed vaginal openings, restoring some of normal anatomy of the genital area, may improve overall reproductive health and function. This necessitates additional investigate in future studies. Consistent with expectations, a reduction in dysmenorrhea and an improvement in sexual quality of life were observed among numerous participants. These outcomes align with the findings of the Nour et al. study conducted on Somali women who had undergone deinfibulation [8]. The decrease in dysmenorrhea implies that partial obstruction of menstrual flow was the primary underlying cause [8].

In the mood of delivery, there has been a slight increase in the number of cesarean sections. This unexpected result can be attributed to the fact that four out of seven participants expressed fear of undergoing another vaginal delivery due to complications associated with female genital mutilation [FGM]. The remaining three participants had previously undergone cesarean sections due to pregnancy-related complications. Interestingly, the participants who opted for vaginal delivery reported that they no longer experienced the same level of complications and difficulties as before. Despite this, the increase in cesarean sections does not align with the findings of a study conducted on multiparous women of African descent in England [19].

Unlike the study of S.A. Raouf and Rouzi [19,20], the rate of obstructed labor, episiotomy, blood loss, and perineum tears decreased, which indicates that deinfibulation is beneficial and has positive effects on obstetric outcomes as we expected. The reason is likely to be that deinfibulation allows for save passaging of the fetal head during delivery [9]. Blood loss during delivery decreased by eleven percent. The reason for blood loss before deinfibulation is likely to be due to episiotomy, in addition to the complications of delivery [21].

More than 24% of the participants no longer suffer from sexual symptoms and have shown improvement in their sexual health. This finding is supported by Nour et al.‘s study [8]. The majority of the remaining participants did not report any changes. Although it was expected that a larger percentage would exhibit improvement, it is speculated that many participants may have withheld information due to the sensitive nature of topics such as female genital mutilation and sexuality in African societies, which are often considered taboo and not openly discussed [8].

It was noted that the urinary tract infection was chronic and recurrent in most cases. However, a small percentage of individuals no longer experience this condition, while the rest have reported a reduction in its frequency. Binkova et al.‘s study provides support for this finding, in which many participants, especially those with FGM type III, reported that genitourinary or sexual symptoms decreased or resolved after their first vaginal birth, which was the moment when deinfibulation or perineal tear happened, widening the vaginal introitus and uncovering the urethral meatus [22]. The reason is likely that the deinfibulation facilitates bladder emptying, thereby lessening the risk of urinary retention and chronic recurrent UTI associated with infibulation [23]. In contrast, Effa et al. did not find any evidence supporting the use of deinfibulation to improve urologic complications in women with type III FGM [24]. The results of the present study were not in line with the results of the above study.

More than half of the participants indicated that the primary reason for not performing the reinfibulation was the experience of pain and fear, as they perceived it as a replication of the traumatic ordeal of FGM. Over 25% of the participants said the reason was risk, particularly among the younger generation who had been educated about the detrimental effects of FGM. They believed that while they had no say in the decision to undergo FGM in the past, they now had the autonomy to protect themselves from the associated health complications. A minority of participants mentioned the unavailability of a midwife as a hindrance to reinfibulation. Additionally, some individuals highlighted that reinfibulation was not a part of their traditions, specifically among the Ben-Rashid and Falata tribes, as well as certain families.

The most common reason for deinfibulation is vaginal delivery, a procedure carried out for every infibulated woman in the labor room. The World Health Organization [WHO] advocates for deinfibulation during delivery and, emphasizes the importance of training nurses and midwives in Africa to implement this practice according to the established standard of care [25,26]. Other reasons for deinfibulation are dyspareunia and apareunia.

Potential confounding factors in the population studied include language barriers, miscommunication, and stigma towards deinfibulation. Also, such studies would have demanded a sample size that was not available to us since most women who underwent deinfibulation for delivery had been reinfibulated again [27]. This highlights the challenge of obtaining suitably large sample sizes. Another difficulty was obtaining accurate responses regarding sexual symptoms, as questions about sexual life may not be culturally appropriate. Another limitation of this study is the absence of detailed, systematic data in the existing literature on specific tribes, their FGM practices, and their demographic contributions. This highlights a significant research gap, underscoring the need for future studies to explore the sociocultural dimensions of FGM practices in greater depth.

This aspect had not been previously discussed in Sudan, where studies primarily focused on reinfibulation and female genital mutilation [FGM] as problems without considering deinfibulation as a potential solution. Therefore, our study offers a unique perspective on this topic.

It is important to note that issues related to female genital mutilation, sexuality, and deinfibulation are typically considered taboo in our culture and are seldom openly discussed. Despite the extreme sensitivity surrounding this topic in Sudanese society, we were able to engage in meaningful discussions and gather sufficient data to draw our conclusions.

### Conclusions

Deinfibulation can improve the quality of life of women who have undergone infibulation before and has positive effects on the urogenital system. It is recommended for all infibulated women, whether modified or total, who suffer from long-term complications such as dysmenorrhea, irregular menses, chronic urinary infection, dyspareunia, apareunia, and delivery complications.

Further initiatives should be undertaken to eradicate FGM through the utilization of various channels, including the media, community influencers, and policymakers. Additionally, a significant number of women who have undergone infibulation may not be informed about the option of deinfibulation to alleviate the complications associated with the procedure, hence, there is a need for community awareness campaigns.

### Supporting information

S1 Data Dataset used for analysis. (CSV)

S1 Text Study questionnaire. (DOCX)

## References

[1] Female genital mutilation [Internet]. World Health Organization; [cited 2024 Jun 24]. Available from: https://www.who.int/news-room/fact-sheets/detail/female-genital-mutilation

[2] Female Genital Mutilation [FGM] statistics - UNICEF data 2024 [Internet]. [cited 2024 Jun 24]. Available from: https://data.unicef.org/topic/child-protection/female-genital-mutilation/.

[3] Female genital mutilation in Sudan - factsheet [Internet]. [cited 2024 Dec 2]. Available from: https://www.unicef.org/sudan/media/9386/file/FGM%20Factsheet-FINAL.pdf

[4] Toubia N. Female circumcision as a public health issue. N Engl J Med. 1994;331(11):712–6. doi: 10.1056/NEJM199409153311106 8058079

[5] Erian MM, Goh JT. Female circumcision. Aust N Z J Obstet Gynaecol. 1995;35(1):83–5. doi: 10.1111/j.1479-828x.1995.tb01838.x 7772009

[6] Mccaffrey M. Female genital mutilation: consequences for reproductive and sexual health. Sex Marital Ther. 1995;10(2):189–200. doi: 10.1080/02674659508405549 12347119

[7] Penna C, Fallani MG, Fambrini M, Zipoli E, Marchionni M. Type III female genital mutilation: clinical implications and treatment by carbon dioxide laser surgery. Am J Obstet Gynecol. 2002;187(6):1550–4. doi: 10.1067/mob.2002.127597 12501062

[8] Nour NM, Michels KB, Bryant AE. Defibulation to treat female genital cutting: effect on symptoms and sexual function. Obstet Gynecol. 2006;108(1):55–60. doi: 10.1097/01.AOG.0000224613.72892.77 16816056

[9] Toubia N. Female genital mutilation and the responsibility of reproductive health professionals. Int J Gynaecol Obstet. 1994;46(2):127–35. doi: 10.1016/0020-7292(94)90227-5 7995451

[10] Female genital mutilation and its management [green-top guideline no. 53] [Internet]. [cited 2024 Jun 23]. Available from: https://www.rcog.org.uk/guidance/browse-all-guidance/green-top-guidelines/female-genital-mutilation-and-its-management-green-top-guideline-no-53/.

[11] WHOQOL - measuring quality of life| The World Health Organization [Internet]. World Health Organization; [cited 2024 Jun 24]. Available from: https://www.who.int/toolkits/whoqol

[12] Kassala State [Internet]. Wikimedia Foundation; 2024 [cited 2024 Jun 23]. Available from: https://en.wikipedia.org/wiki/Kassala_State

[13] Saudi hospital in Kassala - Wikimapia [Internet]. [cited 2024 Jun 24]. Available from: https://wikimapia.org/4952766/Saudi-Hospital-in-Kassala

[14] UNICEF, Unicef. Child marriage in Sudan [Internet]. 2021 [cited 2024 Jul 21]. Available from: https://www.unicef.org/sudan/reports/child-marriage-sudan

[15] Federal Ministry of Health. Taskforce to develop Sudan nursing and midwifery strategy 2012–2016. Final report. Khartoum: Republic of Sudan Federal Ministry of Health; 2011.

[16] Weak law forbidding female genital mutilation in Red Sea state, Sudan [Internet]. [cited 2024 Jun 24]. Available from: https://www.cmi.no/publications/6229-weak-law-forbidding-female-genital-mutilation-in

[17] Sami IR. Female circumcision with special reference to the Sudan. Ann Trop Paediatr. 1986;6(2):99–115. doi: 10.1080/02724936.1986.11748419 2425727

[18] Satti A, Elmusharaf S, Bedri H, Idris T, Hashim MSK, Suliman GI, et al. Prevalence and determinants of the practice of genital mutilation of girls in Khartoum, Sudan. Ann Trop Paediatr. 2006;26(4):303–10. doi: 10.1179/146532806X152827 17132295

[19] Raouf SA, Ball T, Hughes A, Holder R, Papaioannou S. Obstetric and neonatal outcomes for women with reversed and non‐reversed type III female genital mutilation. Intl J Gynecol Obste. 2011;113(2):141–3. doi: 10.1016/j.ijgo.2010.10.028 21306710

[20] Rouzi AA, Al-Sibiani SA, Al-Mansouri NM, Al-Sinani NS, Al-Jahdali EA, Darhouse K. Defibulation during vaginal delivery for women with type III female genital mutilation. Obstet Gynecol. 2012;120(1):98–103. doi: 10.1097/AOG.0b013e3182590343 22914397

[21] Shandall AA. Circumcision and infibulation of females: a general consideration of the problem and a clinical study of the complications in Sudanese women. Sudan Med J. 1967;5(4):178–212. 12259304

[22] Binkova A, Uebelhart M, Dällenbach P, Boulvain M, Gayet-Ageron A, Abdulcadir J. A cross-sectional study on pelvic floor symptoms in women living with Female Genital Mutilation/Cutting. Reprod Health. 2021;18(1):39. doi: 10.1186/s12978-021-01097-9 33581732 PMC7881631

[23] Momoh C, Ladhani S, Lochrie DP, Rymer J. Female genital mutilation: analysis of the first twelve months of a Southeast London specialist clinic. BJOG. 2001;108(2):186–91. doi: 10.1111/j.1471-0528.2001.00036.x 11236119

[24] Effa E, Ojo O, Ihesie A, Meremikwu MM. Deinfibulation for treating urologic complications of type III female genital mutilation: A systematic review. Int J Gynaecol Obstet. 2017;136 Suppl 1:30–3. doi: 10.1002/ijgo.12045 28164295

[25] Management of pregnancy, childbirth and the postpartum period in the presence of female genital mutilation: Report of a who technical consultation, Geneva, 15–7 October 1997 [Internet]. World Health Organization; 1970 [cited 2024 Jun 24]. Available from: https://iris.who.int/handle/10665/66805

[26] World Health Organization. Female genital mutilation: the prevention and the management of the health complications: policy guidelines for nurses and midwives. Geneva: World Health Organization; 2001. Available from: https://iris.who.int/handle/10665/66858.

[27] Lars A, Sta V. Reinfibulation among women in a rural area in central sudan. Health Care for Women International. 2001;22(8):711–21. doi: 10.1080/073993301753339933

