# Supplementary material for: Empowering change: A cross-sectional study on quality of life in deinfibulated women at Saudi Teaching Hospital, Kassala-Sudan 2023–2024
Source: PLOS Glob Public Health. 2025 Feb 14;5(2):e0004096. doi: 10.1371/journal.pgph.0004096 (PMC11828415; doi:10.1371/journal.pgph.0004096)
Supplement: S1 Text — (DOCX) [file pgph.0004096.s002.docx]

**Questionnaire**:

Demographical Information:

1.Age

2.Residence:

A. rural

B. urban

3.Socialclass:

A. high

B. moderate

C. low

4.Occupation

Gynecological and Obstetric history:

1.FGM type

A. Intermediate (type III)

B. Pharaonic (type IV)

2.How old were you when you were circumcised?

Gynecological and obstetric history and outcome before Deinfibulation:

1.Is your menstrual cycle regular?

A. Regular

B. Irregular

2.Do you experience dysmenorrhea?

A. Yes

B. No

3.The mood of your last delivery before deinfibulation

A. Spontaneous vaginal delivery

B. Cesarean section

4.Did you encounter obstructed labor?

A. Yes

B. No

5.Have you undergone an episiotomy?

A. Yes

B. No

6.Was your pelvic perineum preserved intact?

A. Yes

B. No

7.Have you experienced any significant blood loss (blood transfusion)?

A. Yes

B. No

Gynecological and obstetric history and outcome after Deinfibulation:

1. Is your menstrual cycle regular?

A. Regular

B. Irregular

2. Do you experience dysmenorrhea?

A. Yes

B. No

3. The mood of your delivery after deinfibulation

A. Spontaneous vaginal delivery

B. Cesarean section

4. Did you encounter obstructed labor?

A. Yes

B. No

5. Have you undergone an episiotomy?

A. Yes

B. No

6. Was your pelvic perineum preserved intact?

A. Yes

B. No

7. Have you experienced any significant blood loss (blood transfusion)?

A. Yes

B. No

Sexual symptoms:

Have you experience any of these?

1.Lack of interest

Never- after deinfibulation- before deinfibulation

2.Lack of lubrication

Never- after deinfibulation- before deinfibulation

3.Difficulty becoming aroused

Never- after deinfibulation- before deinfibulation

4.Taking long time to become aroused

Never- after deinfibulation- before deinfibulation

5.Pain or discomfort during sexual activity

Never- after deinfibulation- before deinfibulation

6.Difficulty achieving an orgasm (releasing water)

Never- after deinfibulation- before deinfibulation

UTI :

1.Have you ever experienced a UTI ?

A. Yes

B. No

2.Were you diagnosed by a doctor?

A. Yes

B. No

3.When?

A. Before deinfibulation

B. After deinfibulation

C. both

D. never

4.If both, which was the more frequent?

A. Before deinfibulation

B. After deinfibulation

Deifabulation purpose/ reason behind not preforming reinfabulation:

1.Reason behind not performing reinfibulation

A. Painful

B. Risk

C. No midwife available

D. Not in our tradition

E. Other

2.Deinfibulation purpose

A. Vaginal delivery

B. Difficulty in sexual intercourse

C. Other
